# Autistic Traits, Pragmatic Difficulties, and Adaptive Outcomes in Williams Syndrome: A Systematic Narrative Review

**DOI:** 10.3390/children13060750

**Published:** 2026-05-28

**Authors:** Dimitra V. Katsarou, Eleni E. Kyvrakidou

**Affiliations:** Department of Preschool Education Sciences and Educational Design, University of the Aegean, P.C 85132 Rhodes, Greece; psed23008@aegean.gr

**Keywords:** Williams syndrome, autism spectrum disorder, autistic traits, pragmatic language, social communication, adaptive functioning, neurodevelopmental disorders, adaptive and functional outcomes

## Abstract

**Highlights:**

**What are the main findings?**

- Autistic traits are present in a subset of individuals with Williams syndrome and follow a continuum of expression.
- Pragmatic language difficulties represent a consistent and functionally significant domain of impairment.

**What is the implication of the main finding?**

- Socio-communicative functioning in Williams syndrome appears more heterogeneous than traditionally assumed.
- Multidimensional assessment and targeted intervention approaches are necessary in clinical practice.

**Abstract:**

Background and Objectives: Williams syndrome (WS) is a rare neurodevelopmental genetic condition traditionally described as being associated with a highly sociable behavioral profile. However, growing evidence indicates that this characterization may oversimplify the socio-cognitive phenotype, as some individuals with WS exhibit socio-communicative and pragmatic difficulties that may overlap with ASD-related features, although these difficulties should not be interpreted as autism-specific. The present systematic review aimed to investigate the presence of autistic traits in WS, to synthesize evidence on pragmatic and socio-communicative difficulties, and to explore their association with adaptive functioning and, indirectly, adaptive and functional outcomes. Materials and Methods: This study follows a systematic search and selection process in accordance with PRISMA 2020 guidelines and employs a systematic review with structured narrative synthesis. A systematic search of PubMed, Scopus, and Web of Science was performed up to December 2022, supplemented by grey literature sources. Nine studies met the predefined inclusion criteria. Due to substantial methodological heterogeneity, findings were synthesized using a structured narrative approach. Study quality was evaluated using adapted criteria addressing research design, sample characteristics, measurement tools, and risk of bias. Results: The findings suggest that autistic traits may constitute a potentially clinically relevant, though not universal, aspect of the WS phenotype. Pragmatic language difficulties were consistently reported, particularly in relation to conversational management, social reciprocity, and context-appropriate language use. These difficulties appear to function as a key mechanism linking socio-cognitive characteristics with functional outcomes. Patterns of adaptive functioning showed both distinctions from and overlaps with autism spectrum disorder (ASD), especially in communication domains. Available genetic and molecular evidence points to a possible contribution of additional modifying factors for phenotypic variability, with possible phenotypic overlap between WS and ASD, particularly in pragmatic language and adaptive communication. Conclusions: The evidence supports a multidimensional and spectrum-based conceptualization of socio-communicative functioning in Williams syndrome. Despite strong social motivation, individuals with WS may experience meaningful pragmatic and adaptive challenges, with implications for assessment and intervention. These findings highlight the importance of multidimensional and individualized clinical approaches.

## 1. Introduction

Williams syndrome, also known as Williams–Beuren syndrome, is a rare neuro-developmental genetic disorder typically caused by a heterozygous microdeletion of the chromosomal region *7q11.23* and is associated with a distinctive cognitive, linguistic, and socio-emotional phenotype [1,2,3,4]. Traditionally, the syndrome has been characterized as markedly “hypersocial,” as many individuals with Williams syndrome exhibit a strong tendency toward social approach, heightened friendliness even toward strangers, and increased social motivation compared to other neurodevelopmental groups [2,5,6], along with reduced social anxiety [7]. However, although this characterization is descriptively useful, it does not fully capture the complex sociocognitive profile of the syndrome, as apparent sociability does not necessarily imply functional social competence or effective social understanding [1,8,9].

Recent research has highlighted a more complex and heterogeneous picture, indicating that Williams syndrome is not defined solely by heightened sociability but also by difficulties in social understanding, social reciprocity, and communicative functioning [2,9,10,11,12]. Evidence suggests that a subgroup of individuals with Williams syndrome may present with socio-communicative features that overlap with ASD diagnostic criteria; however, these features are not necessarily autism-specific and may also reflect intellectual disability, pragmatic language impairment, adaptive limitations, or broader neurodevelopmental vulnerability [13,14,15]. These findings challenge earlier assumptions that Williams syndrome and autism occupy opposite ends of the social spectrum, suggesting instead that the two conditions may share overlapping difficulties in social cognition, social communication, and adaptive functioning [11,15,16,17,18]. In this review, the term co-occurrence is preferred when referring to the presence of ASD-like features or ASD diagnoses in WS, unless a study explicitly demonstrates independent etiological mechanisms. The term comorbidity is used only when retained from the terminology of the original studies.

Particular attention has been given to the pragmatic dimensions of language—the use of linguistic abilities in real-life social and communicative contexts—as pragmatic difficulties appear to function as a key mechanism of the linguistic phenotype of Williams syndrome [8,9,19,20]. Although individuals with Williams syndrome often demonstrate relatively strong expressive abilities, verbal fluency, and in some cases an extensive vocabulary, they experience significant challenges in managing conversation, maintaining topic coherence, understanding social rules governing communication, and adapting speech to different communicative contexts [8,15,19,20]. These difficulties have been linked to limitations in social perception, theory of mind, joint attention, and broader social cue processing, suggesting that outward social approach does not necessarily reflect mature social understanding or functionally effective communication [1,9,12,19].

The distinction between social motivation and social effectiveness is therefore crucial. While individuals with Williams syndrome may actively seek social interaction, they often encounter challenges in sustaining, regulating, and adapting interactions to contextual demands [2,8,12,19]. Consequently, pragmatic impairments can lead to misinterpretation of social cues, reduced conversational reciprocity, difficulties in establishing and maintaining stable interpersonal relationships, and increased social vulnerability, despite the outwardly sociable behavior frequently observed in clinical contexts [2,8,19].

At the level of functional outcomes, evidence indicates that adaptive functioning in Williams syndrome presents a complex and uneven profile, characterized by relative strengths alongside significant difficulties in communication, socialization, and daily living skills [10,11,12,21]. Comparative studies with individuals with ASD suggest that, despite clear differences in the outward expression of sociability, there are substantial overlaps in communication abilities and overall adaptive functioning. In some cases, differences appear to relate more to the profile of strengths and weaknesses than to overall impairment [10,11,15,22].

These findings suggest that apparent sociability does not necessarily translate into adaptive competence [2,8,11,12]. Furthermore, recent genetic and molecular studies indicate that phenotypic heterogeneity in Williams syndrome may be influenced by complex biological mechanisms beyond the core *7q11.23* microdeletion, including additional rare genetic variants and modifying factors [6,16,23,24]. These variants may contribute to the emergence of autistic traits or even ASD in some individuals, supporting the view that Williams syndrome exists within a broader continuum of neurodevelopmental heterogeneity rather than as a categorically distinct social phenotype [14,16,24].

Taken together, these findings support a nuanced understanding of socio-communicative functioning in Williams syndrome, characterized by the co-occurrence of heightened social approach, sociocognitive difficulties, pragmatic impairments, and variable adaptive outcomes [1,9,12,17].

The aim of the present systematic review is to investigate in depth the relationship between autistic traits, pragmatic difficulties, and adaptive functioning in Williams syndrome, with particular emphasis on their impact on daily life and adaptive and functional outcomes [11,19,20]. Specifically, the review seeks to examine the extent to which autistic traits or comorbidity with ASD are present in individuals with Williams syndrome, to identify the main pragmatic and social-communication difficulties described in the literature, to explore how autistic traits and pragmatic difficulties are associated with adaptive functioning and quality-of-life outcomes, and to investigate which genetic or biological factors may contribute to the observed heterogeneity [11,13,24].

## 2. Materials and Methods

### 2.1. Review Design

This study followed a systematic search and selection process in accordance with PRISMA 2020 guidelines and employed a structured narrative synthesis approach [25,26,27,28]. The review therefore follows the methodological structure of a systematic review while employing narrative synthesis procedures appropriate for heterogeneous evidence. Due to substantial heterogeneity in study designs, assessment tools, and outcome measures, quantitative synthesis and meta-analysis were not feasible. Therefore, findings were synthesized qualitatively using an evidence-weighted framework [25,26,27,28,29]. The review aimed to investigate autistic traits, pragmatic and socio-communicative difficulties, and their relationship with adaptive functioning in Williams syndrome [9,11,17].

The review protocol was retrospectively registered in PROSPERO (ID1372070) on 17 April 2026. Given the limited number of available studies, heterogeneous evidence sources, including grey literature, were included to improve comprehensiveness and were interpreted with lower evidentiary weight [17,25,29].

### 2.2. Search Strategy

The literature search was conducted across the electronic databases PubMed, Scopus, and Web of Science, which are widely used for retrieving biomedical, psychological, and interdisciplinary literature in systematic reviews [25,26,27].

The search employed combinations of keywords and Boolean operators AND and OR, adapted to the requirements of each database to ensure maximal sensitivity and reproducibility in identifying relevant studies [25,28,29]. Specifically, the following combination of terms was used: (“Williams syndrome” OR “Williams-Beuren syndrome” OR “WBS”) AND (“autistic traits” OR “autism spectrum disorder” OR “ASD”) AND (“pragmatic language” OR “social communication” OR “communication phenotype”) [8,13,19].

The term “systematic review” was employed only as a supplementary filter to identify secondary sources and not as a primary filter, in order to avoid restricting the retrieval of primary studies and to maintain as broad a search as possible [25,26,29].

A supplementary search in Google Scholar was conducted to identify grey literature, including master’s theses and doctoral dissertations, which could provide additional relevant data within this limited and specialized field [25,26,27,29]. The inclusion of grey literature was intended to enhance the comprehensiveness of the review and reduce the risk of overlooking potentially relevant findings not indexed in major databases. However, these sources were categorized separately from peer-reviewed studies and were interpreted with lower evidentiary weight due to the absence of formal peer review [25,26,29].

The search was last conducted in December 2022. A limited number of highly relevant studies published after the original search period were incorporated narratively to contextualize recent developments in the field; however, these studies were clearly distinguished from the formally included systematic review dataset. The following search string was used as a base formulation and adapted to the syntax of each database:

(“Williams syndrome” OR “Williams-Beuren syndrome” OR “WBS”) AND (“autistic traits” OR “autism spectrum disorder” OR “ASD”) AND (“pragmatic language” OR “social communication” OR “communication phenotype”).

No restrictions were applied regarding study design. Filters were limited to English-language publications and human participants, where applicable.

In addition, reference lists of included studies were manually screened to identify further relevant articles. Grey literature was retrieved through Google Scholar using combinations of the same keywords.

The detailed reporting of search strategies and database-specific syntax was intended to enhance reproducibility and allow independent replication of the search process. Given the rarity of Williams syndrome and the specificity of the research question, the limited number of eligible studies reflects the current state of the literature rather than a limitation of the search strategy.

### 2.3. Inclusion and Exclusion Criteria

The inclusion criteria were defined as follows: (a) studies published in English, (b) studies published up to 2022, (c) samples including children, adolescents, or adults with genetically or clinically confirmed Williams syndrome, and (d) examination of at least one of the following domains: autistic traits or a diagnosis of autism spectrum disorder, pragmatic language use, social communication or socio-cognitive skills, adaptive functioning, or genetic and neurobiological mechanisms related to the autistic phenotype in Williams syndrome [5,13,23].

Different types of evidence (empirical studies, narrative reviews, and grey literature) were included due to the limited number of available studies; however, these were explicitly categorized and not synthesized as equivalent forms of evidence. Primary empirical studies constituted the core evidence base of the review, while grey literature and narrative sources were used primarily to contextualize emerging patterns in a limited field of research. Their contribution to the synthesis was weighted according to methodological rigor and publication type [17,25,29].

Studies were excluded if they focused exclusively on other neurodevelopmental disorders without specific reference to Williams syndrome, lacked clear relevance to autistic traits or pragmatic difficulties, or had insufficient methodological description or no available full text [25,29,30].

### 2.4. Study Selection Process

The study selection process followed the stages outlined in the PRISMA 2020 framework, including identification of records, removal of duplicates, screening of titles and abstracts, full-text assessment, and final inclusion of studies meeting the predefined criteria [25,26,27,28]. After completion of the search process, records were screened for duplicates and subsequently evaluated based on title and abstract for relevance to the research questions of the present review [25,27,29]. Articles deemed potentially eligible were then assessed at full-text level, and reasons for exclusion were systematically recorded to ensure transparency and reproducibility of the selection process [25,26,28].

**Figure 1:**
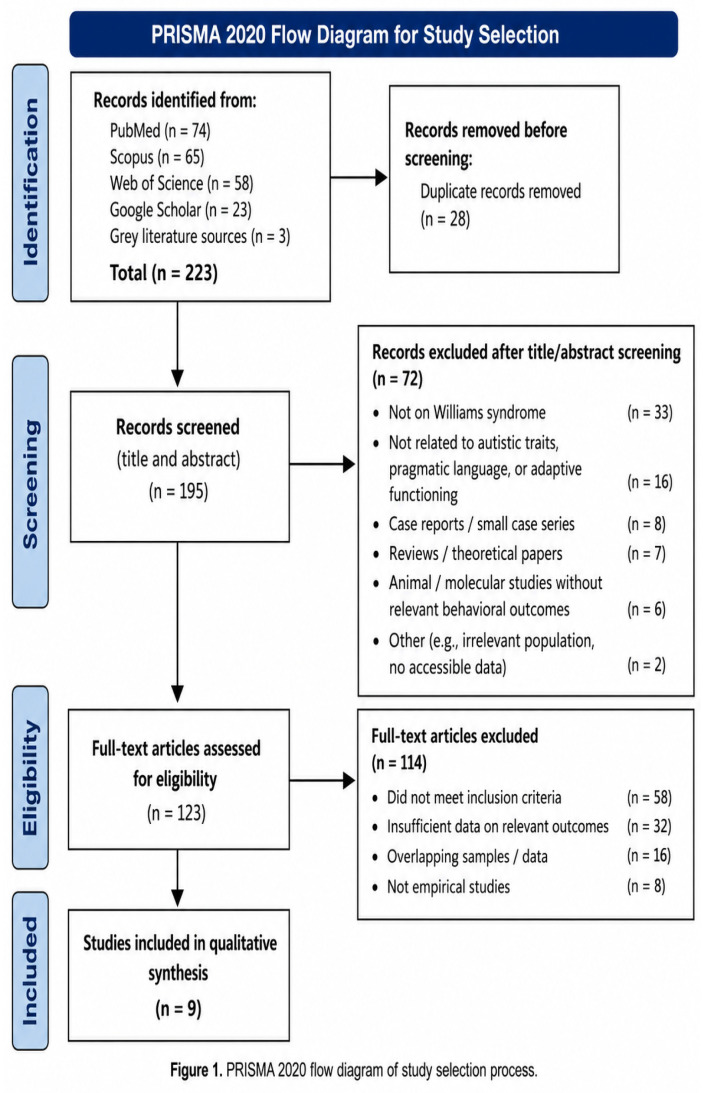
PRISMA 2020 flow diagram illustrating the study selection process, including identification, duplicate removal, screening, eligibility assessment, reasons for exclusion, and final inclusion. Records excluded after title/abstract screening (*n* = 72): unrelated neurodevelopmental disorders (*n* = 21), absence of Williams syndrome sample (*n* = 18), no examination of autistic traits, pragmatics, or adaptive functioning (*n* = 20), conference abstracts/editorials only (*n* = 7), duplicate conceptual reports (*n =* 6).

Study selection and quality assessment were conducted by two reviewers. In cases of uncertainty, the inclusion and exclusion criteria were re-examined and decisions were reached through discussion. Although inter-rater reliability statistics (e.g., kappa coefficients) were not calculated, the use of predefined inclusion criteria and consensus-based decision-making procedures is considered an acceptable approach in systematic narrative reviews of heterogeneous evidence. The final study selection process is presented in Figure 1, following the PRISMA 2020 flow diagram, which illustrates in detail the stages of identification, screening, eligibility assessment, and final inclusion of studies [25,26,27,28]. Despite the structured selection process, the possibility of selection bias cannot be fully excluded, particularly given the limited number of available studies and the inclusion of heterogeneous evidence.

### 2.5. Extraction

For each study, the author and year of publication, study type, sample size and characteristics, assessment tools, main variables, and key findings relevant to the research questions of the review were systematically recorded [25,26,29].

Particular attention was given to distinguishing between direct measurements, such as standardized assessments of autistic traits or pragmatic skills, and indirect indicators of functional impairment, in order to avoid equating non-comparable outcomes within a field characterized by substantial methodological heterogeneity [11,15,19].

### 2.6. Study Quality Assessment

The methodological quality of the included studies was assessed using an adapted appraisal framework based on the Newcastle–Ottawa Scale and Joanna Briggs Institute critical appraisal guidance, in order to accommodate the heterogeneity of included study designs [25,29,30].

Four core domains were evaluated:

(a) Research design (e.g., longitudinal, comparative, observational, or case-series studies),
(b) Sample adequacy and representativeness,
(c) Measurement quality, including the use of standardized and validated instruments, and
(d) Risk of bias, including methodological and reporting limitations [25,29,30].

For empirical studies, sample adequacy was categorized a priori as low (<15 participants), moderate (15–49 participants), or high (≥50 participants), taking into account the rarity of Williams syndrome and conventions commonly used in rare neurodevelopmental research. For narrative reviews and theoretical studies, adequacy was evaluated according to the breadth and transparency of included evidence rather than participant number.

Each domain was qualitatively rated as low, moderate, or high quality, and an overall quality judgment was assigned to each study based on the combined appraisal across domains. Two reviewers independently conducted study selection and quality assessment, and discrepancies were resolved through discussion and consensus.

Studies classified as lower quality or derived from grey literature were not excluded but were interpreted with lower evidentiary weight during the synthesis process. This approach was adopted to enhance transparency and consistency across heterogeneous evidence sources.

### 2.7. Data Synthesis

Due to the substantial methodological heterogeneity of the studies in terms of research designs, assessment tools, sample characteristics, and main variables, quantitative synthesis or meta-analysis was not feasible. Consequently, a narrative synthesis of the findings was adopted [25,26,29].

Where possible, subgroup comparisons across studies (e.g., by study design or outcome domain) were considered; however, due to the limited number of studies and substantial variability in measurement tools and reported outcomes, even partial quantitative aggregation was not methodologically appropriate. As a result, the synthesis relied on a structured qualitative approach incorporating hierarchical weighting and cross-study comparison, rather than numerical aggregation.

The studies were organized into three main thematic areas: (a) autistic traits and comorbidity with autism spectrum disorder in Williams syndrome, (b) pragmatic and socio-communicative difficulties, and (c) the relationship of the above with adaptive functioning and potential genetic or neurobiological mechanisms [13,19,23]. This grouping facilitates a clearer understanding of phenotypic heterogeneity in Williams syndrome and avoids oversimplified interpretations of socio-communicative functioning in Williams syndrome [9,15,17]. Despite efforts to ensure consistency, the application of a unified framework across diverse study designs may introduce limitations in comparability, which should be considered when interpreting quality ratings. A meta-analysis was not feasible due to the substantial heterogeneity in study designs, outcome measures, and assessment tools, which would have compromised the validity of pooled estimates.

#### Analytical Stratification of Evidence

Given the substantial methodological heterogeneity across the included studies, an analytical stratification of evidence was applied to enhance the interpretive rigor of the synthesis. Studies were categorized into hierarchical levels based on research design, methodological robustness, and publication type.

Specifically, the following hierarchy was adopted:

Level 1: Longitudinal and comparative peer-reviewed studies employing standardized assessment tools;
Level 2: Observational studies and clinical case series;
Level 3: Narrative reviews and theoretical studies;
Level 4: Grey literature, including theses and non-peer-reviewed sources.

Greater evidentiary weight was assigned to higher-level studies (Levels 1–2), while findings from lower-level evidence (Levels 3–4) were interpreted with caution and used primarily to support or contextualize emerging patterns. This approach was employed to ensure a more structured and transparent synthesis in the presence of heterogeneous data. Table 1 presents the characteristics of included studies.

## 3. Results

The synthesis of findings was guided by the analytical stratification framework described above, with greater evidentiary weight assigned to higher-level studies. Findings were organized thematically and interpreted through structured comparison across methodological levels, in order to provide an evidence-weighted synthesis of heterogeneous data.

### 3.1. Autistic Traits and Comorbidity with ASD in Williams Syndrome

Studies examining autistic characteristics in Williams syndrome reported variable but clinically relevant ASD-like socio-communicative features across samples [13,14,16,32,33]. Tordjman et al. (2012) [13] described individuals with genetically confirmed Williams syndrome who met diagnostic criteria for ASD based on ADOS and ADI-R assessment. The same research group later reported severe expressive language impairment and autism-related symptomatology in a subgroup of individuals with WS [14].

Willfors et al. (2024) [12] identified elevated autism-related symptoms in a subgroup of participants with Williams syndrome using a transdiagnostic framework. Chichilla (2022) [32], using the Autism Spectrum Rating Scale (ASRS), also reported elevated ASD-related characteristics in children with WS, although this study was based on retrospective and grey-literature methodology.

Genetic and molecular investigations similarly reported overlap between ASD-related and WS-related neurodevelopmental profiles. Codina-Solà et al. (2019) [23] identified additional genetic variants associated with ASD characteristics in individuals with Williams syndrome beyond the classical *7q11.23* deletion. Masson et al. (2019) [16] described six individuals presenting both Williams syndrome and ASD diagnoses, while Niego and Benítez-Burraco (2021) [31] reported similarities in abnormal gene-expression patterns across ASD and WS samples.

At the same time, findings varied substantially according to study design, assessment tools, and sample characteristics. Clinical case-series studies generally reported more severe ASD-related presentations [13,14], whereas larger-scale or retrospective studies more frequently described broader autism-related traits without consistent confirmation of full ASD diagnoses [12,34]. Several studies also included participants with marked intellectual disability, particularly within clinically referred samples [13,14].

Overall, the reviewed studies supported the presence of ASD-like socio-communicative features in a subgroup of individuals with Williams syndrome, although prevalence estimates and clinical severity varied substantially across methodologies, assessment tools, and participant characteristics [12,13,14,16,34].

### 3.2. Pragmatic Difficulties and Social Communication

Pragmatic language difficulties were among the most consistently reported findings across studies examining socio-communicative functioning in Williams syndrome [8,9,15,19,20]. John et al. (2012) [19], in a longitudinal investigation, reported persistent impairments in conversational reciprocity, discourse management, topic maintenance, and context-appropriate language use in children with Williams syndrome.

Additional studies similarly identified difficulties in the pragmatic use of language despite relatively preserved expressive vocabulary and verbal fluency [8,9,15]. Philofsky et al. (2007) [15] found that children with Williams syndrome demonstrated pragmatic-language profiles overlapping with those observed in ASD, particularly in social reciprocity and conversational coherence. Asada et al. (2010) [9] also reported fluent structural language accompanied by impaired pragmatic functioning.

Difficulties in theory of mind, joint attention, and interpretation of social cues were additionally described in several studies examining broader socio-cognitive functioning [17,18,31]. Van der Fluit et al. (2012) [18] reported associations between social-attribution performance and social behavior in Williams syndrome, while theoretical and comparative studies described impairments in social understanding despite high levels of social motivation [17,31].

Family-reported data further supported the presence of pragmatic difficulties affecting everyday communication. Sepúlveda and López Resa (2024) [20] described persistent challenges in conversational functioning and communicative adaptation in daily social interactions.

Across studies, pragmatic impairments were reported in relation to conversational management, social reciprocity, interpretation of communicative context, and adaptive use of language in social interaction [8,9,15,19,20]. Pragmatic impairment emerged as the most consistently replicated finding across methodological levels. Table 2 presents this thematic synthesis of findings across studies.

### 3.3. Relationship with Adaptive Functioning and Adaptive and Functional Outcomes

Several studies examined the relationship between socio-communicative functioning and adaptive outcomes in individuals with Williams syndrome [10,11,21]. Alfieri et al. (2021) [10] and Alfieri et al. (2022) [11] compared adaptive functioning between children with Williams syndrome and children with ASD using the Vineland Adaptive Behavior Scales (VABS). These studies reported uneven adaptive profiles in WS, with relative strengths in sociability but persistent difficulties in communication and daily living skills.

Mervis and Klein-Tasman (2000) [21] similarly described adaptive-functioning variability in Williams syndrome, particularly in relation to communication and social functioning. Additional studies suggested that socio-communicative difficulties may affect social participation, peer relationships, and functional independence [11,35].

Although adaptive and functional outcomes were not directly measured in most studies, several reports described associations between pragmatic communication difficulties, social vulnerability, and challenges in interpersonal functioning [11,20,35]. Gillooly et al. (2024) [35], for example, reported friendship-related difficulties from both parent and child perspectives in children with Williams syndrome.

Overall, the reviewed studies indicated that pragmatic and socio-communicative difficulties were associated with functional and adaptive challenges across multiple domains of daily life. Across studies, adaptive difficulties were more consistently associated with socio-communicative impairment than with social motivation alone.

### 3.4. Genetic and Biological Factors

Several studies investigated genetic and molecular mechanisms potentially associated with phenotypic heterogeneity in Williams syndrome [6,16,23,31]. Codina-Solà et al. (2019) [23] identified additional genetic variants associated with ASD-related symptomatology in individuals with WS beyond the primary chromosomal deletion. Masson et al. (2019) [16] reported heterogeneous molecular findings in individuals presenting both Williams syndrome and ASD diagnoses using chromosomal microarray and whole-exome sequencing approaches.

Niego and Benítez-Burraco (2021) [31] examined gene-expression patterns and reported overlap between molecular pathways associated with ASD and Williams syndrome. Crespi and Procyshyn (2017) [6] also discussed broader genetic mechanisms potentially associated with social behavior, autism-related traits, and neurodevelopmental variability in Williams syndrome.

Across studies, genetic and molecular findings suggested heterogeneous biological contributions to socio-communicative variability within the Williams syndrome phenotype.

Sample adequacy was categorized as low (<15 participants), moderate (15–49 participants), or high (≥50 participants), taking into account the rarity of Williams syndrome and conventions commonly used in rare neurodevelopmental research. For reviews and theoretical studies, sample adequacy was evaluated based on breadth and transparency of included evidence rather than participant number. Measurement quality reflects the use of standardized and validated instruments. Risk of bias was evaluated according to study design, methodological transparency, and publication type. Overall quality reflects combined appraisal across domains.

The overall synthesis of the findings indicates that autistic characteristics in Williams syndrome appear to represent a clinically relevant dimension of the WS phenotype; however, they are not expressed uniformly across individuals. Their presence appears to follow a continuum of expression, reflecting the broader neurodevelopmental heterogeneity of the syndrome.

The evidence suggests that difficulties in the social use of language and in understanding communicative contexts are associated with adaptive functioning and, indirectly, key aspects of adaptive and functional outcomes. Several molecular and genetic studies suggest that variability in socio-communicative presentation in Williams syndrome may be influenced by additional genetic and neurobiological factors beyond the primary *7q11.23* deletion [16,17,23]. However, current evidence remains preliminary and should not be interpreted as establishing a unified etiological pathway between Williams syndrome and ASD. Overall, the findings support a heterogeneous and spectrum-based profile of socio-communicative functioning in Williams syndrome. It should also be noted that part of the supporting evidence includes studies of varying methodological rigor, including non-peer-reviewed sources, which limits the strength of conclusions that can be drawn.

In contrast, evidence regarding the prevalence and clinical significance of autistic traits remains more variable and strongly dependent on study design, sample characteristics, and methodological quality. Therefore, conclusions should be interpreted within the context of these limitations. Therefore, Table 3 presents quality appraisal of included studies.

### 3.5. Evidence-Weighted Narrative Synthesis

Based on the analytical stratification framework, stronger evidence was identified for pragmatic and socio-communicative difficulties than for categorical ASD diagnoses in Williams syndrome. Level 1 evidence, particularly longitudinal and comparative studies such as Alfieri et al. (2022) [11] and John et al. (2012) [19], consistently supported the presence of pragmatic impairments involving conversational reciprocity, discourse management, and adaptive social communication.

Similarly, Level 1 and Level 2 studies demonstrated uneven adaptive-functioning profiles characterized by relative sociability alongside persistent communication and daily-living difficulties [10,11,21]. Across methodological levels, pragmatic impairment emerged as the most consistently replicated finding.

In contrast, evidence regarding autistic traits and ASD diagnoses was more methodologically variable. Level 2 clinical case-series studies, particularly Tordjman et al. (2012) [13] and Tordjman et al. (2013) [14], reported clinically significant ASD-related features in subsets of individuals with Williams syndrome. However, broader prevalence estimates were derived primarily from Level 4 grey-literature or retrospective studies, including Chichilla (2022) [32], and therefore require more cautious interpretation.

Overall, the synthesis supports stronger and more consistent evidence for pragmatic and socio-communicative difficulties than for categorical ASD comorbidity in Williams syndrome. Pragmatic impairments were replicated across methodological levels and were consistently associated with adaptive and functional outcomes [8,9,15,19,20]. In contrast, evidence regarding ASD diagnoses remained methodologically variable and dependent on study design and sample characteristics [12,13,14,34].

## 4. Discussion

The present review examined autistic traits, pragmatic difficulties, and adaptive functioning in Williams syndrome within a limited but clinically important body of literature. Although the available evidence remains methodologically variable, the findings consistently suggest that heightened sociability in Williams syndrome does not necessarily imply intact socio-communicative functioning [2,8,12,19]. Rather, the reviewed studies support a more variable phenotype in which strong social motivation may coexist with significant difficulties in social understanding, conversational reciprocity, and adaptive communication [8,9,15,19].

One of the most important findings concerns the presence of ASD-like socio-communicative features in a subgroup of individuals with Williams syndrome [12,13,14,16]. Importantly, these findings should not be interpreted as indicating diagnostic equivalence between Williams syndrome and ASD. Instead, they suggest phenotypic overlap in domains such as pragmatic language, social reciprocity, and adaptive communication across neurodevelopmental conditions [11,17,31].

Pragmatic difficulties constitute a clinically important domain of impairment in Williams syndrome. These findings further emphasize the distinction between social motivation and functional communicative competence.

The relationship between pragmatic difficulties and functional outcomes also constitutes a critical finding. Although research in this area remains limited, the available evidence suggests that socio-communicative dysfunction appears closely associated with adaptive functioning. Alfieri et al. (2022) suggested that individuals with Williams syndrome, despite certain strengths in socialization, do not exhibit overall better functional adaptation compared to individuals with ASD [11]. These findings, although informative, remain constrained by indirect measurement and variability in study design. Finally, genetic and molecular studies support the view that the heterogeneity of the Williams syndrome phenotype has a multifactorial basis. The findings indicate that the classical microdeletion alone is insufficient to explain the presence of autistic characteristics, and that additional genetic or modifying factors may play a critical role [16,23]. This observation aligns with a broader framework that conceptualizes neurodevelopmental disorders as overlapping spectra with potentially overlapping neurodevelopmental mechanisms.

The findings of this review have direct clinical implications. Specifically, they highlight the need for systematic assessment of pragmatic skills and autistic characteristics in individuals with Williams syndrome, regardless of apparent sociability. The traditional view that these individuals are socially “protected” may lead to underdiagnosis and delayed intervention. Furthermore, the evidence supports the development of targeted interventions focusing on social understanding, conversational reciprocity, and functional communication. Within these constraints, the present review contributes to clarifying patterns of convergence across heterogeneous studies, while also identifying key gaps that require more methodologically rigorous investigation. The present review integrates heterogeneous evidence through an explicit hierarchical weighting framework. This approach allows a more structured and nuanced interpretation of autistic traits, pragmatic difficulties, and adaptive functioning across methodological levels. A limitation of the current evidence is that most studies emphasize social communication and pragmatic language, whereas restricted interests and repetitive behaviors are less consistently examined. This is important because repetitive behaviors may occur in WS and other intellectual disability syndromes without necessarily indicating ASD. Future studies should distinguish repetitive behaviors associated with intellectual disability or syndrome-specific profiles from those that form part of a broader ASD presentation [36,37,38].

## 5. Conclusions

The present review suggests that pragmatic and socio-communicative difficulties represent important dimensions of the Williams syndrome phenotype, while ASD-like features may occur in a subgroup of individuals [12,13,14,15,19]. Overall, the findings support a complex and heterogeneous socio-communicative profile in Williams syndrome [9,15,17]. Despite heightened social motivation, many individuals with Williams syndrome experience clinically relevant difficulties in social understanding, conversational reciprocity, and adaptive communication, with important implications for assessment and intervention [8,11,19].

These findings further suggest the need for a multi-level approach to assessment and intervention that considers both the overt social characteristics and the less visible socio-cognitive difficulties. These conclusions should be interpreted as reflecting the current state of evidence rather than definitive characterization, and remain subject to revision as more robust empirical data become available.

## 6. Study Limitations

This review has several limitations that should be considered when interpreting the findings. First, the available evidence base remains limited and methodologically heterogeneous, including longitudinal studies, comparative investigations, clinical case series, molecular genetic studies, narrative reviews, and selected grey literature [25,26,29]. This variability complicated direct comparison across studies and precluded quantitative synthesis or meta-analysis.

Second, considerable variability existed in the assessment tools used to evaluate autistic characteristics, pragmatic language, social communication, and adaptive functioning across studies. Different methodological approaches and outcome measures may partly explain inconsistencies in the reported findings [11,15,19].

Third, quality of life was rarely assessed directly using standardized measures. Consequently, conclusions regarding quality of life are inferred indirectly through adaptive functioning, social participation, and interpersonal outcomes rather than through direct quality-of-life assessments [11,20,35].

Fourth, the inclusion of grey literature, including master’s theses and doctoral dissertations, enhanced comprehensiveness within this limited field but was interpreted with lower evidentiary weight due to the absence of peer review [25,26,27,29].

Fifth, the review was restricted to English-language publications, which may have resulted in the exclusion of potentially relevant studies published in other languages.

Additionally, although the review followed PRISMA 2020 guidelines [25,28], the available body of evidence did not allow for highly homogeneous comparisons or strong causal conclusions. Furthermore, the PROSPERO registration was conducted retrospectively after data extraction and synthesis had been completed, which may reduce methodological transparency and increase the risk of selective reporting.

Another limitation is that several included studies did not adequately control for intellectual functioning. This is important because lower cognitive ability may contribute to pragmatic-language difficulties, adaptive impairments, repetitive behaviors, and elevated ASD screening scores. Consequently, some apparent overlap between Williams syndrome and ASD may partly reflect broader developmental vulnerability or intellectual disability rather than autism-specific mechanisms [38].

Despite these limitations, the review contributes to clarifying patterns of socio-communicative functioning across heterogeneous studies and highlights important directions for future research in Williams syndrome.

## 7. Recommendations for Future Research and Clinical Practice

Future research would benefit from focusing on larger, multicenter, and longitudinal studies in order to capture more accurately the prevalence, presentation, and developmental trajectory of autistic characteristics in Williams syndrome. Given the rarity of the syndrome, the development of collaborative research networks across centers and countries could substantially contribute to increasing sample sizes and enhancing the external validity of findings.

Particular emphasis should be placed on the use of common and standardized assessment tools, in order to enhance comparability of results across research groups and cultural contexts. Future studies should more clearly distinguish between autistic characteristics, pragmatic difficulties, socio-cognitive skills, and broader adaptive functioning, to avoid conceptual overlap between distinct yet related constructs.

Furthermore, research is needed that examines pragmatics and social communication as discrete and central domains, rather than merely secondary aspects of linguistic or social functioning. A more detailed characterization of pragmatic deficits could substantially contribute to understanding the mechanisms through which apparent sociability coexists with functional difficulties in communication and interpersonal interaction.

Equally important is the inclusion of direct and standardized measures of quality of life in future research, both for individuals with Williams syndrome and for their families. This approach would enable a more comprehensive understanding of how socio-communicative difficulties affect not only functional outcomes but also subjective well-being, social participation, and long-term adaptation.

At the same time, there is a particular need for studies linking behavioral and clinical findings with genetic, neurobiological, and developmental mechanisms. Further investigation of potential modifying factors could contribute to a better understanding of phenotypic heterogeneity and support more precise models of risk and prognosis.

At the clinical level, the findings of this review indicate that individuals with Williams syndrome should not be assumed to be inherently protected from autism-like difficulties solely because of their heightened social approach. Instead, systematic and multidimensional assessment of autistic characteristics, social reciprocity, pragmatic language use, social understanding, and adaptive functioning is warranted.

Interventions need to be individualized and should not be limited to enhancing language production or social motivation. Rather, it is important that they include targeted support for understanding social rules, managing conversational reciprocity, interpreting social and nonverbal cues, and generalizing communicative skills to everyday life. In this context, collaboration among speech–language therapists, psychologists, child psychiatrists, educators, and families is of critical importance.

Finally, from an educational and clinical-organizational perspective, recognizing the heterogeneity of Williams syndrome underscores the need to develop early detection protocols, assessment guidelines, and interdisciplinary intervention models that take into account not only the overtly positive social characteristics but also the less visible socio-cognitive and pragmatic difficulties.

## Figures and Tables

**Table 1:** Characteristics of Included Studies.

Study (Author, Year)	Evidence Level	Design	Sample	Age Group	Measures/Tools	Main Focus
John et al., 2012 [19]	Level 1	Longitudinal	N = 14 (WS)	Children	Pragmatic language assessments; discourse analysis	Pragmatic functioning
Alfieri et al., 2021 [10]	Level 1	Comparative cross-syndrome	WS vs. ASD	Children	Vineland Adaptive Behavior Scales (VABS)	Adaptive profile
Alfieri et al., 2022 [11]	Level 1	Longitudinal comparative	WS vs. ASD	Children	VABS	Adaptive functioning
Tordjman et al., 2012 [13]	Level 2	Clinical case series	N = 9 (WS)	Children/adolescents	ADOS; ADI-R	ASD-related features
Tordjman et al., 2013 [14]	Level 2	Clinical case series	N = 2 (WS + ASD)	Children/adolescents	Clinical assessment; serotonin biomarkers	Severe ASD features
Codina-Solà et al., 2019 [23]	Level 2	Genetic study	WS with ASD traits	Not specified	Whole-exome sequencing; CNV analysis	Genetic modifiers
Masson et al., 2019 [16]	Level 2	Molecular genetic study	N = 6 (WS + ASD)	Not specified	Chromosomal microarray; WES	Molecular overlap
Niego & Benítez-Burraco, 2021 [31]	Level 2	Molecular comparative study	Not applicable	Not applicable	Gene-expression analysis	Molecular overlap
Philofsky et al., 2007 [15]	Level 1	Comparative study	WS vs. ASD	School-age children	Pragmatic language assessment	Pragmatic profile
Van der Fluit et al., 2012 [18]	Level 1	Observational	N = 26 (WS)	Children/adolescents	Social cognition tasks	Social cognition
Sepúlveda & López Resa, 2024 [20]	Level 2	Family-report observational	Families of individuals with WS	Mixed	Family-reported pragmatic skills	Everyday communication
Chichilla, 2022 [32]	Level 4	Retrospective grey literature study	N = 180 (WS)	Children	Autism Spectrum Rating Scale (ASRS)	ASD symptom profile
Codina i Solà, 2016 [24]	Level 4	Doctoral thesis	Not specified	Not specified	Genetic analysis	Genetic variation
Niego & Benítez-Burraco, 2020 [17]	Level 3	Narrative review	Not applicable	Not applicable	Literature synthesis	Socio-cognitive overlap

WS = Williams syndrome; ASD = Autism Spectrum Disorder; ADOS = Autism Diagnostic Observation Schedule; ADI-R = Autism Diagnostic Interview-Revised; VABS = Vineland Adaptive Behavior Scales; WES = Whole Exome Sequencing; CNV = Copy Number Variation. Ratings were assigned based on predefined criteria described in Section 2.6.

**Table 2:** Thematic synthesis of findings across studies.

Thematic Domain	Main Findings	Representative Studies	Strength of Evidence
Autistic traits and ASD-like features	ASD-like socio-communicative features identified in a subgroup of individuals with WS; prevalence varied across studies	Tordjman et al., 2012 [13]; Tordjman et al., 2013 [14]; Willfors et al., 2024 [12]; Chichilla, 2022 [32]	Moderate
Pragmatic difficulties	Persistent difficulties in conversational reciprocity and pragmatic communication	John et al., 2012 [19]; Philofsky et al., 2007 [15]; Asada et al., 2010 [9]	Strong
Social cognition and socio-communicative functioning	Difficulties in social understanding and interpretation of social cues	Van der Fluit et al., 2012 [18]; Vivanti et al., 2016 [22]; Niego & Benítez-Burraco, 2020 [17]	Moderate
Adaptive functioning	Uneven adaptive profile with persistent communication and daily-living difficulties	Alfieri et al., 2021 [10]; Alfieri et al., 2022 [11]; Mervis & Klein-Tasman, 2000 [21]	Moderate–Strong
Quality of life and social participation	Socio-communicative difficulties associated with social vulnerability and reduced participation	Gillooly et al., 2024 [35]; Sepúlveda & López Resa, 2024 [20]	Limited–Moderate
Genetic and biological mechanisms	Genetic and molecular factors may contribute to phenotypic variability	Codina-Solà et al., 2019 [23]; Masson et al., 2019 [16]; Niego & Benítez-Burraco, 2021 [31]	Moderate

Sample adequacy refers to sample size and representativeness. Measurement quality reflects the use of standardized instruments. Risk of bias was assessed based on study design. Overall quality reflects combined evaluation.

**Table 3:** Quality appraisal of included studies.

Study (Author, Year)	Evidence Level	Design	Sample Adequacy	Measurement Quality	Risk of Bias	Overall Quality
John et al., 2012 [19]	Level 1	Longitudinal	Low (<15 participants)	High (standardized measures)	Moderate	Moderate
Alfieri et al., 2021 [10]	Level 1	Comparative cross-syndrome	Moderate	High (VABS)	Low	High
Alfieri et al., 2022 [11]	Level 1	Longitudinal comparative	Moderate–High	High (VABS)	Low	High
Tordjman et al., 2012 [13]	Level 2	Clinical case series	Low (<15 participants)	High (ADOS; ADI-R)	Moderate	Moderate
Tordjman et al., 2013 [14]	Level 2	Clinical case series	Very low	Moderate–High	High	Low–Moderate
Codina-Solà et al., 2019 [23]	Level 2	Genetic study	Moderate	High (advanced molecular methods)	Low–Moderate	High
Masson et al., 2019 [16]	Level 2	Molecular genetic study	Low	High	Moderate	Moderate
Niego & Benítez-Burraco, 2021 [31]	Level 2	Molecular comparative	Indirect/secondary evidence	High	Moderate	Moderate–High
Philofsky et al., 2007 [15]	Level 1	Comparative study	Moderate	High	Moderate	Moderate–High
Van der Fluit et al., 2012 [18]	Level 1	Observational	Moderate	High	Moderate	Moderate
Sepúlveda & López Resa, 2024 [20]	Level 2	Family-report observational	Moderate	Moderate	Moderate	Moderate
Chichilla, 2022 [32]	Level 4	Retrospective grey literature	High	Moderate (rating scale only)	High	Moderate
Codina i Solà, 2016 [24]	Level 4	Doctoral thesis	Not specified	Moderate	High	Moderate
Niego & Benítez-Burraco, 2020 [17]	Level 3	Narrative review	Indirect/secondary evidence	Moderate	High (review-level bias)	Moderate

## Data Availability

Data sharing is not applicable to this article, as no new datasets were generated or analyzed. The review protocol was retrospectively registered in PROSPERO (ID1372070).
